# Congenital muscular dystrophy-associated inflammatory chemokines provide axes for effective recruitment of therapeutic adult stem cell into muscles

**DOI:** 10.1186/s13287-020-01979-y

**Published:** 2020-11-02

**Authors:** Vitali Alexeev, Jacquelyn Olavarria, Paolo Bonaldo, Luciano Merlini, Olga Igoucheva

**Affiliations:** 1 Department of Dermatology and Cutaneous Biology, Jefferson Medical College, Thomas Jefferson University, 233 South 10th Street, BLSB, Rm. 430, Philadelphia, PA 19107 USA; 2 Departments of Molecular Medicine, University of Padova, Padova, Italy; 3 Department of Biomedical and Neuromotor Sciences, University of Bologna, Bologna, Italy

**Keywords:** Type VI collagen, COL6A1, Congenital muscular dystrophy, Adipose-derived stem cell, Cell-based therapy, Chemokines, Chemotaxis

## Abstract

**Background:**

Congenital muscular dystrophies (CMD) are a clinically and genetically heterogeneous group of neuromuscular disorders characterized by muscle weakness. The two most prevalent forms of CMD, collagen VI-related myopathies (COL6RM) and laminin α2 deficient CMD type 1A (MDC1A), are both caused by deficiency or dysfunction of extracellular matrix proteins. Previously, we showed that an intramuscular transplantation of human adipose-derived stem cells (ADSC) into the muscle of the *Col6a1*^*−/−*^ mice results in efficient stem cell engraftment, migration, long-term survival, and continuous production of the collagen VI protein, suggesting the feasibility of the systemic cellular therapy for COL6RM. In order for this therapeutic approach to work however, stem cells must be efficiently targeted to the entire body musculature. Thus, the main goal of this study is to test whether muscle homing of systemically transplanted ADSC can be enhanced by employing muscle-specific chemotactic signals originating from CMD-affected muscle tissue.

**Methods:**

Proteomic screens of chemotactic molecules were conducted in the skeletal muscles of COL6RM- and MDC1A-affected patients and CMD mouse models to define the inflammatory and immune activities, thus, providing potential markers of disease activity or treatment effect. Also using a pre-clinical animal model, recapitulating mild Ullrich congenital muscular dystrophy (UCMD), the therapeutic relevance of identified chemotactic pathways was investigated in vivo*,* providing a basis for future clinical investigations.

**Results:**

Comprehensive proteomic screens evaluating relevant human and mouse skeletal muscle biopsies offered chemotactic axes to enhance directional migration of systemically transplanted cells into CMD-affected muscles, including CCL5-CCR1/3/5, CCL2-CCR2, CXCL1/2-CXCR1,2, and CXCL7-CXCR2. Also, the specific populations of ADSC selected with an affinity for the chemokines being released by damaged muscle showed efficient migration to injured site and presented their therapeutic effect.

**Conclusions:**

Collectively, identified molecules provided insight into the mechanisms governing directional migration and intramuscular trafficking of systemically infused stem cells, thus, permitting broad and effective application of the therapeutic adult stem cells for CMD treatment.

## Background

Alterations of extracellular matrix (ECM) or dysfunction of ECM-associated proteins have been implicated in a variety of diseases, notable among those are muscular dystrophies. Congenital muscular dystrophies (CMD) are a clinically and genetically heterogeneous group of neuromuscular disorders characterized by muscle weakness within the first 2 years of life [1]. The two most prevalent forms of CMD, i.e., collagen VI (COL6)-related myopathies (COL6RM) and laminin α2 (LAMA2)-deficient CMD type 1A (MDC1A), share a similar underlying disease mechanism, consisting in the deficiency or dysfunction of extracellular matrix (ECM) proteins. Both disorders are unique among other hereditary myopathies and considered hybrid disorders with clinical features attributed to both muscle and connective tissue and often-called disorders of myomatrix. Treatment options for CMD patients are limited and mainly focused on addressing the clinical manifestations [2]. Due to the distinct cellular origin of the proteins involved in the common CMD forms, even though a range of therapeutic approaches have been tested for traditional muscular dystrophies, there is a need to develop treatment strategies that specifically target muscle ECM alterations.

Adult stem cell-based therapy emerged as a promising strategy for treating genetic diseases, including different types of muscular dystrophies, in particular, Duchenne muscular dystrophy [3, 4]. The majority of studies evaluated the ability of muscle-derived stem cells or progenitor cells, such as myoblasts, side population cells, myogenic endothelial cells, pericytes, and mesoangioblasts, as well as cells from non-muscle tissues to replace damaged muscle fibers [5–9]. However, transplanted cells showed limited ability to regenerate muscle fibers despite the production of missing proteins by transplanted cells but showed reduction of inflammation through trophic effects elicited by transplanted cells.

Effective cell therapy of ECM-related CMD such as COL6RM and MDC1A rests on the ability of the therapeutic cells to secrete normal ECM proteins that can prevent muscle cell degeneration rather than on the potential of stem cells to differentiate into muscle fibers. Also, since the primary defect of COL6RM and MDC1A are in muscle ECM but not in muscle cells the ability of transplanted cells to differentiate into muscle cells is not crucial and secondary to their function of supplying the missing extracellular components. Until recently, bone marrow was considered the main source for adult stem cells. Recent advancements in stem cell isolation protocols allowed discovery alternative repository of stem cells such as subcutaneous fat. Similarly, to bone marrow-derived mesenchymal stem cells, adipose-derived stem cells (ADSC) can be obtained by less invasive methods, differentiate into multiple linages, have relatively low donor-site morbidity, and are available in large quantities for procurement. Also, ADSC have potential as immunoprivileged universal cells with capacity for the allogeneic transplantation and reduced possibility of developing graft-versus-host disease. On special note, ADSC produce a significant level of COL6 and to less extent LAMA2 proteins, making these cells readily available for therapeutic intervention of CMD after local and systemic administration [10].

Previously, we obtained a proof-of concept data showing that human neonatal ADSC delivered intramuscularly can participate in restoration of COL6 deficiency in mouse model of COL6RM [10]. We have found that transplantation of the xenogeneic ADSC leads to efficient engraftment of adult stem cells into the interstitial connective tissue of the skeletal muscles, long-term survival of stem cells up to 3 months in muscle environment, and continuous production of therapeutic the COL6 protein. These findings suggested the possibility of a durable clinical benefit following cell transplantation and provided the scientific rationale for developing stem cell therapy for CMD.

Effective therapy requires the delivery of therapeutic stem cells to all body musculature, a problem that cannot be easily overcome unless systemic transplantation protocols are proved to be effective. Previous studies showed that systemic transplantation of bone marrow- and muscle-derived stem cells had a limited impact on muscle cell replacement and improvement of murine muscular dystrophy, partially due to poor cell recruitment to the muscle tissue. Also, it is known that a large fraction of systemically infused stem cells are passively trapped in draining organs, such as lungs and liver [11]. For the same token, the relative heterogeneity of stem cells, the limited repertoire of functional chemokine receptors on stem cell surface and possibly other factors impede effective homing of stem cells to the skeletal muscle tissue [12–14].

Currently, molecular mechanisms governing recruitment of systemically transplanted stem cells from circulation to the skeletal muscle are largely unknown. However, prior studies have demonstrated that skeletal muscles after experimental injury and dystrophic muscles of *mdx* mice are characterized by an inflammatory “molecular signature” in which CC and CXC chemokines are prominent [15, 16]. The key mechanism that regulates cell recruitment to distal anatomical sites and migration of cells inside affected tissue is chemotaxis, which depends on the signaling molecules termed chemokines. In the adult organism, expression of most chemokines is induced in response to physiologic stress or damage. Being secreted, chemokines recruit leukocytes, progenitor stem cells, and other cell types to the diseased/damaged sites as part of host defense and repair mechanism. In physiological conditions, diverse insults provoking muscle damage and repair trigger rapid and significant leukocyte recruitment to the affected muscles through activation of chemotactic factors produced by muscle cells as wells as other resident cells such as macrophages, known to be dominant generators of chemokines and inflammatory and growth factors [17]. In turn, the produced factors draw in inflammatory cells by chemotaxis to clear debris and further promote growth and differentiation of myoblasts and endothelial smooth muscle cells required for tissue regeneration. In case of genetic dystrophies such as CMD, sustained muscle fiber damage during muscle contractions leads to fiber degeneration accompanied by inflammation and immune cell infiltration. It is well known that a prominent feature of CMD-affected muscle is a striking inflammatory infiltrate of immune cells such as macrophages, neutrophils, and T and B cells [18, 19]. Based on current knowledge of leukocyte trafficking, it is plausible that systemically transplanted ADSC can be recruited from circulation to the CMD muscles by the activation of specific receptors on the surface of transplanted ADSC, followed by transmuscular migration mediated by stem cell receptors under influence of the CMD muscle-derived chemokines. Through characterization of specific chemokines secreted by CMD-damaged muscles and delivery of stem cells with activated receptors to those chemokines, therapeutic stem cells can potentially be delivered to sites of disease-affected muscles and arrest further damage to compromised muscle tissue.

In the current study, we performed comprehensive proteomic screens of chemotactic molecules in the skeletal muscles of COL6RM- and MDC1A-affected patients and CMD mouse models and identified chemokine receptor axes available for recruitment of stem cells to the CMD-affected skeletal muscles. Then, selected stem cell populations were tested for their ability to promote disease-driven chemotaxis-based muscle homing in vivo using pre-clinical mouse model of COL6RM. Results of this investigation could provide avenue for the potential clinical treatment of CMD and related muscular disorders.

## Methods

### Mouse strains

All animal procedures were performed in accordance with the “Guide for the Care and Use of Laboratory Animals” (National Institute of Health publication no. 86–23) and approved by the Institutional Animal Care and Use Committee of the Thomas Jefferson University. Wild-type C57BL/6 mice were purchased from The Jackson Laboratory (Bar Harbor, ME, USA). NCr nude spontaneous mutant **(**CrTac:NCr-*Foxn1*^*nu*^) (Taconic, Derwood, MD, USA). *DyW* (B6.129S1(Cg)-*Lama2*^tm1Eeng^) mice were purchased from The Jackson Laboratory (Bar Harbor, ME, USA). This model has markedly reduced expression of a truncated LAMA2 chain and displays severe muscular dystrophy and peripheral neuropathy. *DyW* newborn mice begin to show evidence of disease by two weeks of age, are passive, small, and emaciated, and demonstrate partial hind-leg lameness and clasping and die by approximately 7 weeks of age. *Col6a1*^*−/−*^ mice were previously described [20]. *Col6a1*^*−/−*^ possess a disruption of the gene encoding the α1(VI) collagen chain, resulting in the lack of COL6 secretion. The phenotype of the *Col6a1*^*−/−*^ mice is mild. C*ol6a1*^*−/−*^ mice have normal life span and somewhat smaller size than the wild-type animals.

### Muscle biopsies from CMD-affected patients

Muscle biopsies from healthy individuals were purchased from ProteoGenex, Inc. (Culver City, CA, USA). Muscle biopsies from CMD patients were obtained from Congenital Muscular Dystrophy Tissue Repository (CMD-TR, Milwaukee, WI, USA) and Dr. Luciano Merlini (Bologna, Italy) according to ethical committee rules and country’s regulations. No patient-identifiable information was available to researchers. The study was not determined as human subject research as per guidelines of the Thomas Jefferson University IRB and as such did not require ethics approval. Clinical features of the patients are presented in Table S1.

### Tissue biopsies from CMD mouse models

The gastrocnemius muscle (GCM) was collected from *dyW* and wild-type C57BL/6 mice at week one through 7 weeks of age, respectively. For each time point, biopsies from five animals were pooled and processed for the total protein isolation as described previously [10]. Due to the absence of significant muscle damage or inflammation in the mild-phenotype *Col6al* ^*−/−*^ mice, muscle damage was induced by administration of 10 μM cardiotoxin (CTX, Sigma, St. Louis, MO, USA) to the right GCM of the 6-month-old *Col6a* ^*−/−*^ and wild-type mice, respectively. Animals were sacrificed at day 0 (baseline), 1, 3, 7, 14, and 21 days post-CTX treatments. Biopsies of GCM from 5 animals in each cohort were pooled and processed for total protein isolation.

### Chemokine antibody arrays

Human Chemokine Antibody Arrays (RayBioTech, Norcross GA, USA) and Proteome Profiler™ Mouse Chemokine Antibody Array (R&D Systems, Minneapolis, MN, USA) were used to assay skeletal muscle biopsies from patients affected with Bethlem myopathy, UCMD, MDC1A, and mouse tissues derived from CMD mouse models, *dyW* and *Col6al* ^*−/−*^ mice, respectively. Five hundred micrograms of total protein were used to probe the chemokine antibody array according to the manufacturer’s instructions. Chemokine antibody array membranes were developed by standard enhanced chemiluminescence techniques as advised by manufacturers. Acquisition of signals on chemokine arrays was quantitatively determined using ScanAlize version 2.50 (Stanford University) and GEArray Expression Analysis Suite 2.0 software (SABiosciences, Frederick, MD, USA), which reads the images and matches them to the corresponding protein on the array. The net level of each protein was calculated by the mean of the individual spot intensity minus the mean of the background intensity. To provide normalization, the average level ratio of two principal proteins was determined and introduced as a correction factor. Relative spot intensities are presented as mean ± SD. Microsoft Excel (Microsoft, Redmond, WA, USA) was utilized for statistical analysis.

### Isolation of mouse adipose-derived stem cells and tissue culture conditions

Mouse subcutaneous fat was excised and maintained on ice. Standard method for isolating and purifying ADSC from the stromal vascular fraction (SVF) containing fibroblasts, pericytes, preadipocytes, monocytes, and macrophages, as well as smooth muscle, endothelial progenitor, and red blood cells of the SVF, have been well established and was employed here [10, 21]. Briefly, tissue was digested in warmed DMEM/F12-Glutamax media (Thermo/Fisher, Grand Island, NY, USA) supplemented with 0.1% collagenase (Sigma. St. Louis, MO, USA) and 1% Penicillin/Streptomycin (Thermo/Fisher) while shaking at 37 °C and 180 rpm for 1 h. To obtain a single cell suspension, the digested tissue was applied to a 30-μm mesh separation filter (Miltenyi Biotec, Auburn, CA, USA). PBS+1% BSA solution was added to the mesh to quench the enzyme and flush any remaining cells through the filter. The suspension was centrifuged and the pellet was resuspended in 1 ml of DMEM/F12 and Glutamax+10% FBS (Thermo/Fisher). Cells were plated in DMEM/F12 and Glutamax plus 10% FBS (Invitrogen, Grand Island, NY, USA) and grown to confluence. To remove hematopoietic cells, cells then were depleted using magnetic separation beads (Miltenyi Biotec). Resultant CD31^−^CD45^−^ population defined as ADSC was designated as first passage cells and was cultured in DMEM/F12-Glutamax media supplemented with 10% FBS and penicillin/streptomycin.

### Generation and characterization of Ccr2- and Cxcr2-overexpressing ADSC

Full-length mouse Ccr2 and Cxcr2 receptor with 3′ UTR was amplified from total mouse RNA via reverse transcription reaction using Superscript II RT Kit (Invitrogen, Carlsbad, CA, USA) followed by PCR using PFU II high fidelity polymerase (Agilent Technologies, Santa Clara, CA, USA). Resultant cDNA was inserted into pEF2-TOPO vector. Integrity of the promoter and cDNA was verified by direct DNA sequencing. Minimally cultured ADSC (passage 1–2) were nucleofected with pEF1-mCcr2 and pEF1-mCxcr2 plasmids, respectively, using Lonza nucleofection reaction (T-27 program, nucleofection kit V; Lonza, Cologne, Germany). Further, pool of Ccr2- and Cxcr2-expressing cells was selected with Blasticidin (0.5 mg/ml; Invitrogen) for 10 days, respectively. Expression of Ccr2 and Cxcr2 in selected cells was confirmed by FACS and indirect immunofluorescence analyses. Receptor surface expression was determined by FACS using PE-conjugated antibodies. For indirect immunofluorescence, Ccr2- and Cxcr2-immunocomplexes were detected with Alexa-Fluor^488^-conjugated secondary antibodies (Invitrogen). Nuclei were counterstained with 4′,6-diamidino-2-phenyl indol (DAPI; Sigma, St. Louis, MO, USA). Immunofluorescent images were obtained on Nikon TS100F fluorescent microscope (Nikon, Melville, NY, USA).

### Transplantation of ADSC into mice under physiological and pro-inflammatory conditions

All animal procedures were performed in accordance with the “Guide for the Care and Use of Laboratory Animals” (National Institute of Health publication no. 86-23) and approved by the Institutional Animal Care and Use Committee of the Thomas Jefferson University. For enforced migration of stem cells into skeletal muscle, ADSC uniformly expressing Ccr2 were stably transduced with Luciferase reporter gene. Native and Ccr2-expressing ADSC were systemically administered via tail vein injection into NCr nude mice, respectively, following localized intramuscular administration with the mouse recombinant Ccl2 chemokine into the left GCM. For therapeutic assessment of Ccr2- and Cxcr2-ADSC in physiological and pro-inflammatory conditions, 2.5 × 10^6^ cells were labeled with a red lipophilic tracer DiOC18 (Molecular Probes, Grand Island, NY, USA) and systemically transplanted into 4 month old *Col6a1*^*−/−*^ mice (*n* = 5/treatment/time point) via tail vein injection. To induce pro-inflammatory injury, the left hindlimb was injected with 10 μM cardiotoxin 3 days prior transplantation. The right GCM was injected with PBS and used as a control for natural homing. For analysis, transplanted mice were euthanized by CO_2_ inhalation at predetermined time points (4, 14, and 21 days) and muscle samples were collected. In vivo imaging (IVIS) (Lumina XR, Caliper LifeSciences, Hopkinton, MA, USA) was performed at indicated time points to determine the localization of the transplants. At indicated time points, muscle biopsies were collected, embedded into OCT compound (VWR, Pittsburgh, PA, USA), frozen, and cryosectioned at a thickness of 10 μm. All samples were evaluated for the presence of engrafted DiOC18-ADSC expressing cells using fluorescence microscopy. For indirect immunofluorescence analysis, cross-sections were stained with rabbit polyclonal anti-α1(VI) collagen (kindly provided by Timpl Laboratory) and rat monoclonal anti-LAMA2 (clone 4H8–2; Enzo Life Sciences, Inc., Farmingdale, NY, USA) antibodies. Immunocomplexes were detected with AlexaFluor^488^- and AlexaFluor^594^-labeled secondary antibodies (Invitrogen). Average number of collagen α1(VI)-positive fibers was calculated and compared between samples collected at different time points. Multiple adjacent sections were analyzed within 20 random, non-overlapping microscopic fields per sample. All morphometric comparisons are presented as percentages of untreated limb (baseline control) and analyzed for statistical significance using the Student’s *t* test, with *p* value less than 0.01 considered significant in all tests.

### Statistical analysis

Data are presented as group mean values ± S.E.M. All array data represent the average values obtained from at least three independent experiments performed on separate occasions. Statistical comparisons were performed using Student’s *t* test for independent samples or ANOVA in the case of multiple comparisons. A value of *P* < 0.05 was considered significant.

## Results

### Proteomic screens of chemokines in the skeletal muscles of CMD patients

**Fig. 1:**
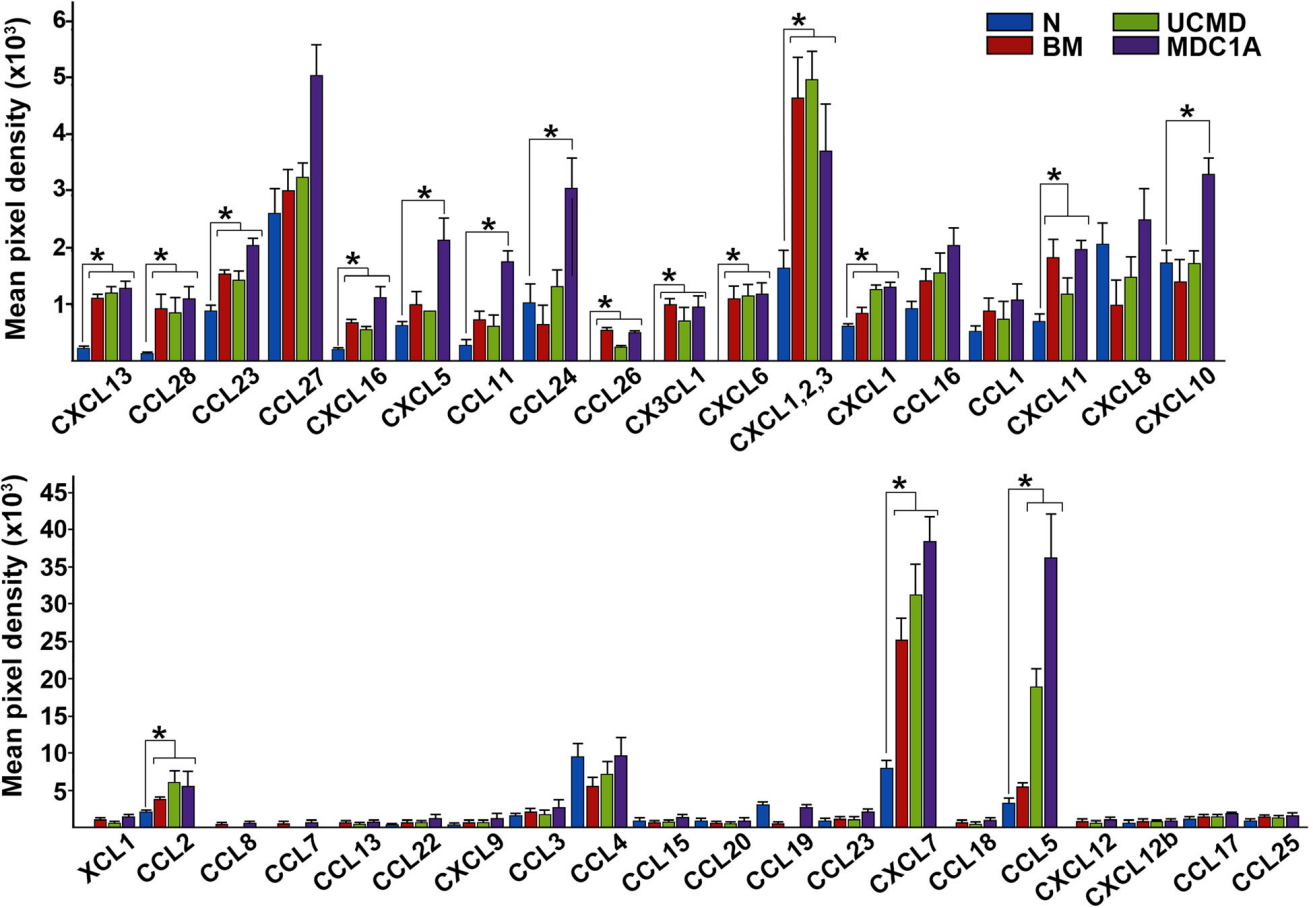
Proteome analysis of chemokines in muscles of CMD-affected patients. The data were collected from independent arrays with duplicate measurements for each chemokine using 5 BM, 8 UCMD, and 5 MDC1A muscle samples (Table S1). Chemokines are listed below the columns. Data are presented as a mean pixel density ± SD. Statistical significance (*p* < 0.05) is indicated by asterisk. CMD types are indicated in the key. N, normal (control); BM, Bethlem myopathy; UCMD, Ulrich congenital muscular dystrophy; MDC1A, Merosin-deficient congenital muscular dystrophy type 1A

Proteome analysis consisted of skeletal muscle biopsies from patients with confirmed diagnosis of Bethlem Myopathy (BM, *n* = 5), Ulrich Congenital Muscular Dystrophy (UCMD, *n* = 8), and Merosin-deficient congenital muscular dystrophy type 1A (MDC1A, *n* = 5). Patients were from 2 to 67 years of age and presented a range of clinical symptoms from mild to severe. The clinical characteristics of CMD patients are summarized in Table S1. Assay controls included skeletal muscle biopsies collected from 18, 43, and 58-year-old healthy individuals with no CMD history. The human chemokine array was used to simultaneously survey 38 known human chemokines. The data were analyzed by grouping the patients by disease type (BM, UCMD and MDC1A) and represented based on a comparison to muscle samples of healthy donors. The ratio of mean pixel densities of individual chemokine in CMD samples to that in control group is presented as the fold difference (Table 1, Fig. S1A). Analysis of biopsies collected from the healthy group demonstrated that homeostatic muscles contain constitutively low levels and limited repertoire of chemokines, with CTAK (CCL27), GRO (CXCL1,2,3), IL-8 and IL-10, MCP-1 (CCL2), MIP-1 beta (CCL4), NAP-2 (CXCL7), and RANTES (CCL5) being above the minimal value of the detection. Proteome profile of CMD-derived biopsies reveled disease-related changes (Fig. 1). Cross analysis of patient data showed that all examined muscle biopsies share similar chemokine profile regardless of genetic abnormality or severity of disease. Moreover, most dominant molecules were found to be a pro-inflammatory chemokines and common for all three CMD types, those included NAP-2 (CXCL7), GCP (CXCL6), GRO (CXCL1,2,3), RANTES2 (CCL5), and MCP-1 (CCL2) (Fig. 1, Table 1). On special note, the level of CCL5 was associated with CMD severity, showing modest increase (1.7-fold) in BM-derived muscles and considerably higher presence in muscles of UCMD (5.7-fold) and MDC1A (11.0-fold) patients (Table 1). However, prognostic value of this molecule will require further statistical analysis in a larger cohort of patients with careful phenotypic evaluation. Collectively, robust analysis of CMD muscle-associated chemokines revealed a distinct subset of chemokines that may contribute to the pathology of the major CMD types. These data also revealed several chemotactic pathways that could be further exploited for the effective recruitment and homing of the systemically transplanted therapeutic stem cells to CMD-affected muscles and the improvement of cell-based therapies.

**Table 1:** Proteome analysis of selected chemokines in muscle biopsies of CMD-affected patients

Chemokine	Fold difference/control^a^		
	BM	UCMD	MDC1A
CCL28	6.6	6.1	7.9
CK beta 8-1	1.7	1.6	2.3
CTACK	1.2	1.2	1.9
CXCL-16	3.2	2.6	5.3
ENA-78	1.6	1.4	3.3
Eotaxin	2.6	2.2	6.2
GCP-2	1099.5	1151.4	1189.1
GRO	2.9	3.1	2.3
GRO-alpha	1.4	2.0	2.1
HCC-4	1.5	1.7	2.2
I-309	1.7	1.4	2.0
I-TAC	2.6	1.7	2.8
IL-8	0.5	0.7	1.2
IP-10	0.8	1.0	1.9
MCP-1	1.8	3.0	2.7
NAP-2	3.3	4.1	5.0
RANTES	1.7	5.7	11.0

^a^Data represented as fold difference of CMD muscle vs healthy (control) muscle. Each protein array was processed in an identical manner and the number represents an average of triplicate experiments from 5 BM, 8 UCMD, and 5 MDC1A muscle samples. Fold difference does not reflect quantity. *BM* Bethlem myopathy, *UCMD* Ulrich congenital muscular dystrophy, *MDC1A* Merosin-deficient congenital muscular dystrophy type 1A

### Proteome analysis of chemokines in LAMA2-deficient mice

**Fig. 2:**
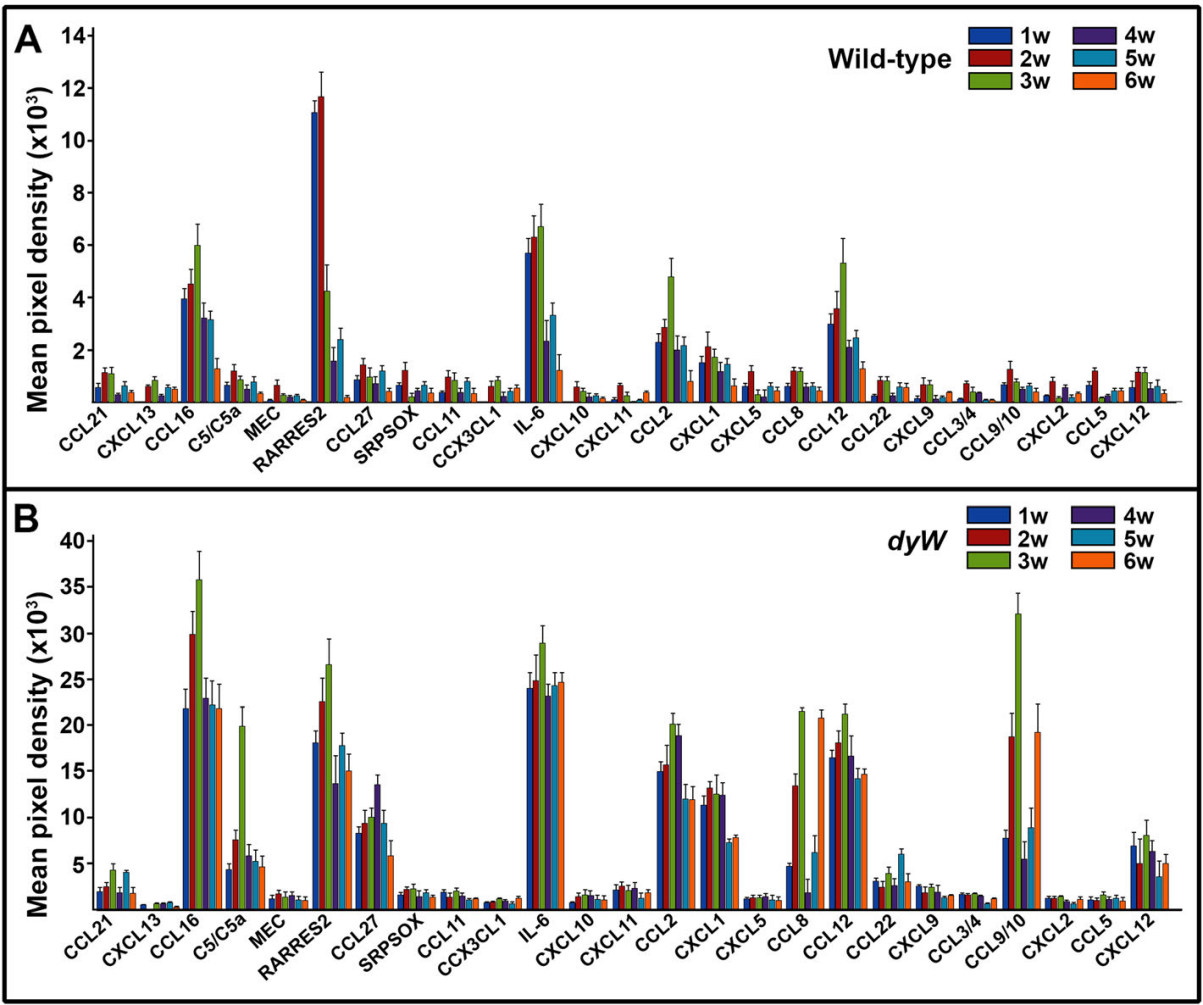
Proteomic screens of chemokines in muscles of *dyW* mice. Data were collected from independent arrays with duplicate measurements for each chemokine using muscle biopsies from wild-type (**a**) and *dyW* (**b**) mice, respectively, at 1, 2, 3, 4, 5, and 6 weeks after birth. Time points are indicated in the key. Chemokines are listed below the columns. Data are presented as a mean pixel density ± SD. w, week(s)

The *Lama2* (*dyW*) mice recapitulate the clinical manifestations of MDC1A patients and display severe muscular dystrophy, with death around 5–7 weeks of age. Proteomic screens of chemotactic molecules in the gastrocnemius muscle (GCM) were conducted at different stages of disease progression: initial (7–14 days), acute (3–4 weeks), and terminal (5–6 weeks). Assay controls were consisted of proteomic profiles utilizing the GCM tissues from age-matched wild-type counterparts. Densitometry data analysis of individual chemokine is reported as the fold difference change of *dyW* compared to wild-type samples. Evaluation of 25 known mouse chemotactic molecules in the GCM of wild-type mice showed constitutive presence of few chemokines at low levels, mainly produced as a part of normal homeostasis of muscle tissue (Fig. 2a, Fig. S1B). Contrary, analysis of the GCM from *dyW* mice showed abundant presence of several chemotactic molecules at all stages of disease (Fig. 2b). Pairwise comparison of the GCM sampled from *dyW* and normal mice revealed the significant induction of seven distinct CC and CXC class chemokine ligands, including CCL6, C5/C5a, RARRES2, CCL27, IL-16, CCL2, CXCL1, CCL8, CCL12, CCL9/CCL10, and CXCL12 (Table 2). Patterns of most identified chemokines showed early induction as soon as 1 week of age and maintained expression until termination point. Analysis of the diaphragm muscle (DM) sampled from *dyW* and wild-type mice showed considerable presence of CCL6 and RARRES2 and modest de-regulation of IL16, CCL2, CXCL1, and CCL12 (data not shown). Interestingly, the levels of some identified molecules were variable depending on the tissue and did not necessarily coincide with stage of disease. Specifically, CCL6, RARRES2, and CCL9/10 showed the highest level at initial stage of disease (2 weeks) in the DM but reached the maximum only in acute phase (3 weeks) in the GCM. For the same token, IL6 level had modest increase at 2 weeks in the DM and was maintained at that level as disease progressed; however, its level did not change significantly and was steady in the GCM. CCL8 and CCL12 levels dis not vary much and demonstrated consistent pattern associated with all disease stages. Contrary, analysis of serum samples did not produce any noticeable changes in chemokine profile despite disease progression; however, the serum collected from 1-week-old animals showed temporal increase in CCL6 (5.6-fold), CXCL5 (4.9-fold), and CCL9/CCL10 (9.0-fold), which reached the basal level by second week of life (data not shown). Together, these data suggest that rapid induction and sustained expression of several chemotactic molecules in the GCM and DM of LAMA2-deficient mice may provide a selective mechanism for inflammatory cell recruitment and, thus, play a role in disease pathology. Also, identified chemotactic signatures suggest that therapeutic stem cells can be recruited to the affected tissues by the similar chemotactic mechanism as immune cell trafficking.

**Table 2:** Proteome analysis of chemokines in muscle biopsies of *dyW* mice

Chemokine	Fold difference/control^a^					
	Time since birth, weeks					
	1	2	3	4	5	6
CCL21	3.4	2.1	3.8	6.0	6.4	4.7
CXCL13	1.5	0.2	0.7	2.7	1.3	0.6
CCL6	5.6	6.6	6.0	7.1	7.0	17.0
C5/C5a	6.8	6.3	23.1	12.3	6.8	14.0
CCL28	1.5	2.6	3.5	3.7	4.0	3.2
RARRES2	1.6	1.9	6.3	8.6	7.4	76.2
CCL27	9.7	6.6	10.3	19.6	8.0	14.2
CXCL16	2.5	1.8	2.5	3.0	2.7	4.1
CCL11	5.1	1.3	2.5	3.8	1.3	3.2
CX3CL1	53.6	1.2	1.4	4.4	1.6	2.2
IL-16	4.2	3.9	4.3	9.9	7.3	20.1
CXCL10	2.6	2.4	4.0	7.9	4.6	6.5
CXCL11	2.1	3.8	2.8	3.7	2.0	4.8
CCL2	6.5	5.5	4.2	9.5	5.5	15.2
CXCL1	7.4	6.3	7.3	10.8	5.1	12.5
CXCL5	2.0	1.0	4.9	6.0	1.7	2.5
CCL8	7.7	11.4	18.3	3.0	4.4	23.5
CCL12	5.5	5.1	4.0	8.0	5.7	11.3
CCL22	12.7	3.0	4.9	9.9	10.5	5.6
CXCL9	3.5	2.6	3.7	4.2	5.2	4.0
CC3/CCL4	14.6	2.2	4.3	3.9	8.5	13.0
CCL9/CCL10	11.5	15.0	41.2	10.6	14.2	47.3
CXCL2	5.4	1.6	8.1	1.5	3.3	3.1
CCL5	1.6	0.9	12.6	4.6	2.6	2.2
CXCL12	11.7	4.3	7.0	12.1	6.0	14.1

^a^Data represented as fold difference of *dyW* muscle vs muscle from age-matched wild-type counterpart (control). Each protein array was processed in an identical manner and the number represents an average of triplicate experiments from 5 pooled muscle biopsies. Fold difference does not reflect quantity

### Proteome analysis of chemokines in COL6-deficient mice under physiological and pro-inflammatory conditions

**Fig. 3:**
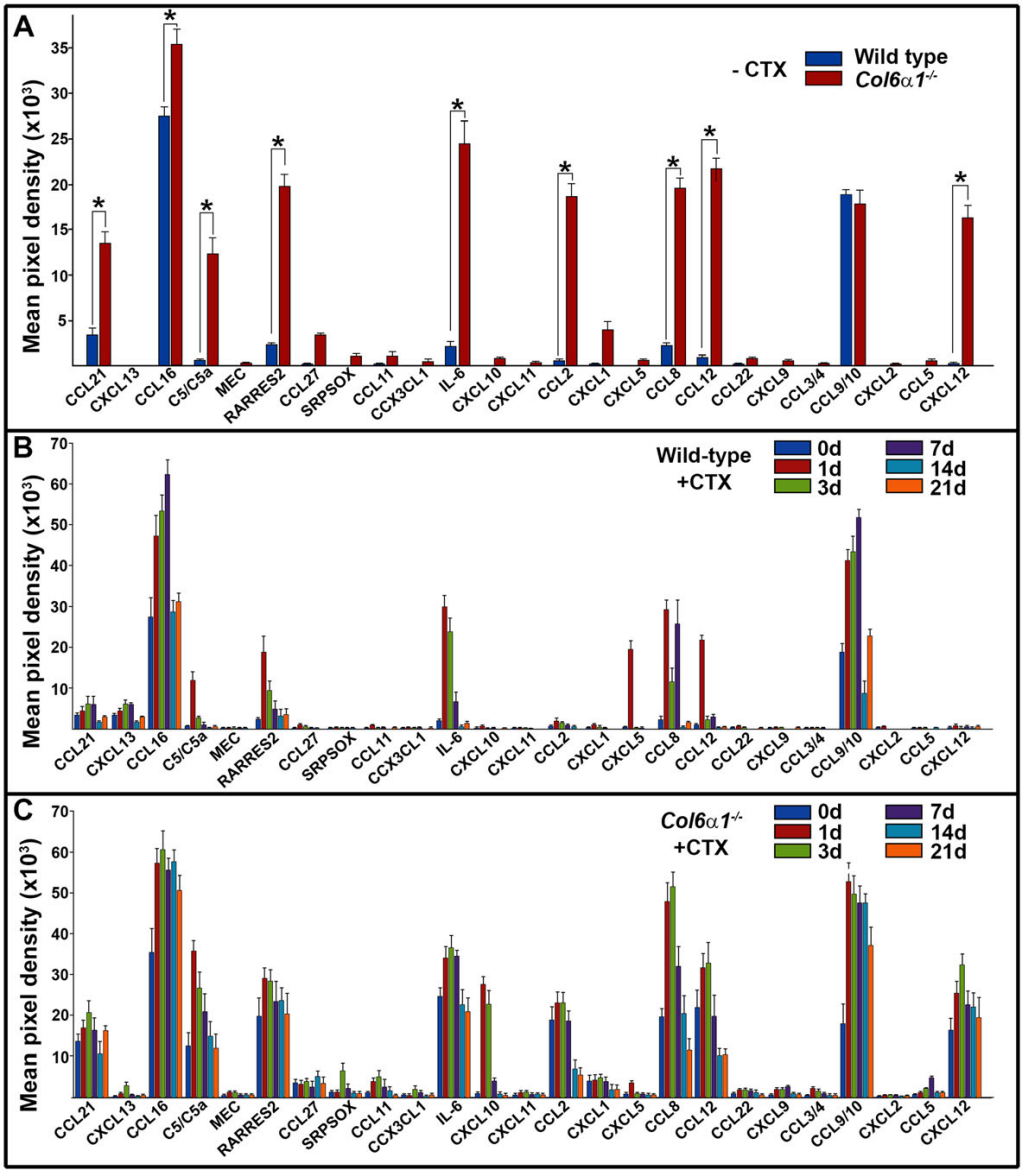
Proteomic screens of chemokines in muscles of *Col6a1*^*−/−*^ mice under physiological and pro-inflammatory conditions. **a** Data were collected from independent arrays with duplicate measurements for each chemokine using muscle biopsies from wild-type and *Col6a1*^*−/−*^ mice, respectively, under physiological conditions (−CTX). **b**, **c** Data were collected from independent arrays with duplicate measurements for each chemokine using muscle biopsies from wild-type (**b**) and *Col6a1*^*−/−*^ (**c**) mice, respectively, under pro-inflammatory conditions (+CTX). Post-injury time points (days) are indicated in the key. Chemokines are listed below the columns. Data are presented as a mean pixel density ± SD. Statistical significance (*p* < 0.05) is indicated by asterisk. d, day(s)

The homozygous *Col6a1*^*−/−*^ mutants completely lack COL6 in the tissues but have normal life span. Skeletal muscles of adult *Col6a1*^*−/−*^ mice display a myopathic histology, including fiber necrosis, phagocytosis, a pronounced variation in the fiber diameter, and signs of stimulated regeneration of fibers with necrotic fibers particularly frequent in the diaphragm [20]. Since the muscle phenotype of the *Col6a1*^*−/−*^ mice is much milder than that of the human UCMD patients, CTX was used to exacerbate the temporal muscle abnormality, as previously shown in this model [22]. CTX selectively injures myofibers but leaves nerves, blood vessels, and satellite cells morphologically intact. Proteome profile was undertaken to define a signature of chemokine release corresponding to sequential stages of skeletal muscle injury and regeneration. Following CTX treatment, the GCM biopsies were harvested at defined periods (0/uninjured, 1, 3, 7, 14, and 21 days). Assay controls consisted of data collected from the age-matched wild-type mice under similar treatment conditions. Data is presented as the fold-change difference of densitometry reads between the *Col6a1*^*−/−*^ and control groups. Comparative proteome profiling of the *Col6a1*^*+/+*^ and *Col6a1*^*−/−*^ GCMs under uninjured conditions showed considerable presence of several chemokines in *Col6a1*^*−/−*^*-*derived muscles, including Ccl21 (4.0-fold), C5/C5a (18.1-fold), RARRES2 (8.4-fold), IL16 (11.9-fold), Ccl2 (29.3-fold), Ccl8 (8.7-fold), Ccl12 (22.3-fold), Cxcl1 (5-fold), and Cxcl12 (42.6-fold) (Fig. 3a and Table 3). Twenty-four hours after CTX injury, a substantial release of several pro-inflammatory chemokines was evident in the wild-type GCM, as judged by increased levels of Ccl6, C5/C5a, RARRES2, IL16, Cxcl5, Ccl8, Ccl12, and Ccl9/Ccl10 (Fig. 3b). Interestingly, chemokine profile of *Col6a1*^*−/−*^ mice showed modest response to CTX, affecting only a few molecules, including C5/C5a, Cxcl1, Cxcl10, Ccl8, and Ccl9/Ccl10 (Fig. 3c). Moreover, the level of induced chemokines was comparable between wild-type and *Col6a1*^*−/−*^ muscles, with the exception of Ccl21, C5/C5a, Cxcl10, and Cxcl12 (Table 3). Further analysis of regenerating muscles showed that the levels of CTX-induced chemokines in wild-type GCM were reestablished to the baseline values by 2 weeks after injury. In contrast, high levels of CTX-induced chemokines in *Col6a1*^*−/−*^ mice persisted for 2 weeks until they restored to the basal level by week 3 (Table 3).

**Table 3:** Proteome analysis of chemokines in muscle biopsies of *Col6a1*^*−/−*^ mice

Chemokine	Fold difference/control^a^					
	Time after CTX treatment, days					
	0	1	3	7	14	21
Ccl21	4.0	3.8	3.3	2.7	6.3	5.5
Cxcl13	1.1	1.1	1.1	1.1	1.1	1.1
Ccl6	1.3	1.2	1.1	0.9	2.0	1.6
C5/C5a	18.1	3.0	9.6	19.9	80.2	23.0
CCL28	1.1	1.2	1.1	1.2	1.1	1.3
RARRES2	8.4	1.5	3.0	4.8	7.7	6.0
Ccl27	13.9	3.2	8.3	5.5	6.0	3.0
SRPSOX	1.1	1.2	4.8	1.1	1.2	1.1
Ccl11	1.1	1.2	1.1	1.1	1.2	1.1
Cx3cl1	1.1	1.1	1.1	1.1	1.1	1.1
IL16	11.9	1.1	1.5	5.1	36.4	14.6
Cxcl10	10.5	39.6	45.6	10.2	1.1	1.1
Cxcl11	1.1	1.1	1.1	1.1	1.1	1.1
Ccl2	29.3	12.2	15.4	18.9	13.1	16.7
Cxcl1	1.2	1.1	1.1	1.2	1.1	1.2
Cxcl5	1.2	4.3	1.1	1.2	1.1	1.2
Ccl8	8.7	1.6	4.4	1.2	48.7	7.5
Ccl12	22.3	1.5	14.7	6.8	35.9	20.9
Ccl22	1.1	1.1	1.2	1.1	1.2	1.1
Cxcl9	1.1	1.1	1.2	1.1	1.2	1.1
Ccl3/Ccl4	1.1	1.1	1.2	1.1	1.2	1.1
Ccl9/Ccl10	0.9	1.3	1.1	0.9	5.4	1.6
Cxcl2	1.2	1.1	1.2	1.1	1.2	1.1
Ccl5	1.1	1.1	1.2	1.1	1.2	1.1
Cxcl12	42.6	38.4	182.8	47.7	45.6	47.7

^a^Data represented as fold difference of *Col6a1*^*−/−*^ muscle vs muscle from age-matched wild-type counterpart (control). Each protein array was processed in an identical manner and the number represents an average of triplicate experiments from 5 pooled muscle biopsies. Fold difference does not reflect quantity

### Chemotaxis-mediated recruitment of ADSC to skeletal muscles in vivo

**Fig. 4:**
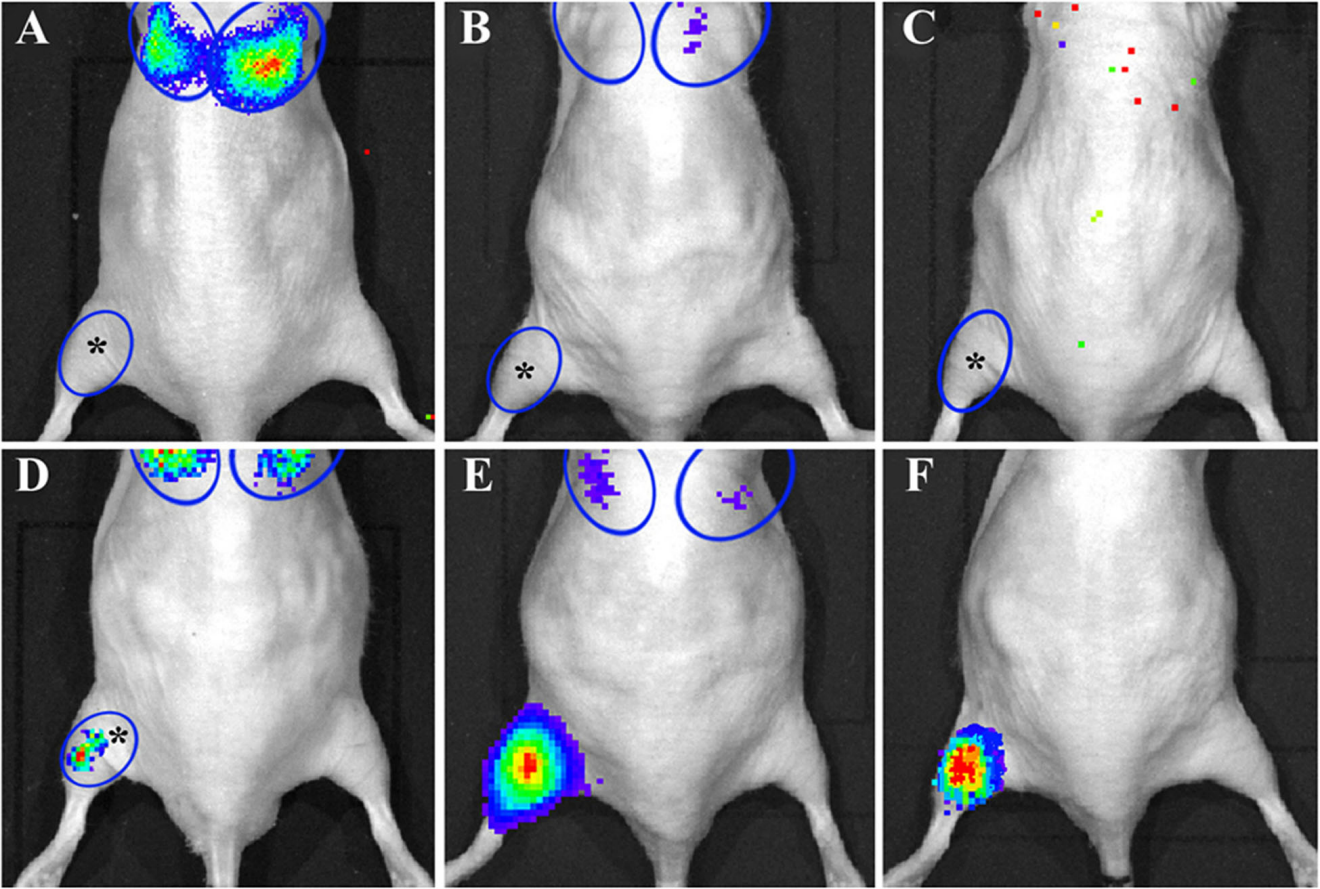
Representative live imaging of Ccl2/Ccr2-mediated recruitment of systemically transplanted heterogeneous ADSC (**a**, **c**, **e**) and Ccr2-positive ADSC (**b**, **d**, **f**) into the GCM of NCr nude mice. Mouse recombinant Ccl2 chemokine was administered into the left GCM immediately after cell transplant. Cell recruitment to the muscle was assessed by luciferase fluorescence at 24, 48, and 72 h after transplantation, respectively. Oval demarcates luciferase-ADSC fluorescence. Asterisk in oval indicates Ccl2 chemokine injection site

Our previous FACS-based assessment of receptor activity in minimally cultured primary stem cells showed that ADSC have very limited percentage of cells with functional chemokine receptors, accounting for their ineffective recruitment into muscles [21]. Because homing of systemically infused ADSC to the muscle could be enhanced via the use of cells uniformly expressing principal receptor, ADSC engineered to express Ccr2 were systemically transplanted into NCr nude mice followed by localized intramuscular administration of the mouse recombinant Ccl2 chemokine into the left GCM. As expected, mice transplanted with heterogeneous ADSC (less than 6% of cells positive for Ccr2 receptor) showed significant cell entrapment in lungs during first 24 h, with no detectable engraftment into chemokine-treated or untreated limbs (Fig. 4a). In the next 48–72 h, control mice did not show any appreciable signals, indicating exiting of cells along unspecific axes within the body (Fig. 4b, c). In sharp contrast, as early as 24 h after transplantation, migration of Ccr2-postive ADSC along created chemotactic gradient was observed in the chemokine-treated GCM but not in untreated right limb (Fig. 4d). Additional 48 h led to a more robust migration of the receptor-expressing cells from the circulation to the chemokine-treated muscle, as revealed by the marked increase in red fluorescent signal (Fig. 4e, f). Direct immunofluorescence analysis of muscle biopsies showed preferential colonization of Ccr2-positive ADSC around blood vessels (data not shown). Taken together, these findings strongly support our hypothesis that engagement of stem cell receptor with tissue-derived chemokine is a critical step in cell recruitment into muscle tissue.

### Chemotactic recruitment of ADSC to CMD-affected muscles

**Fig. 5:**
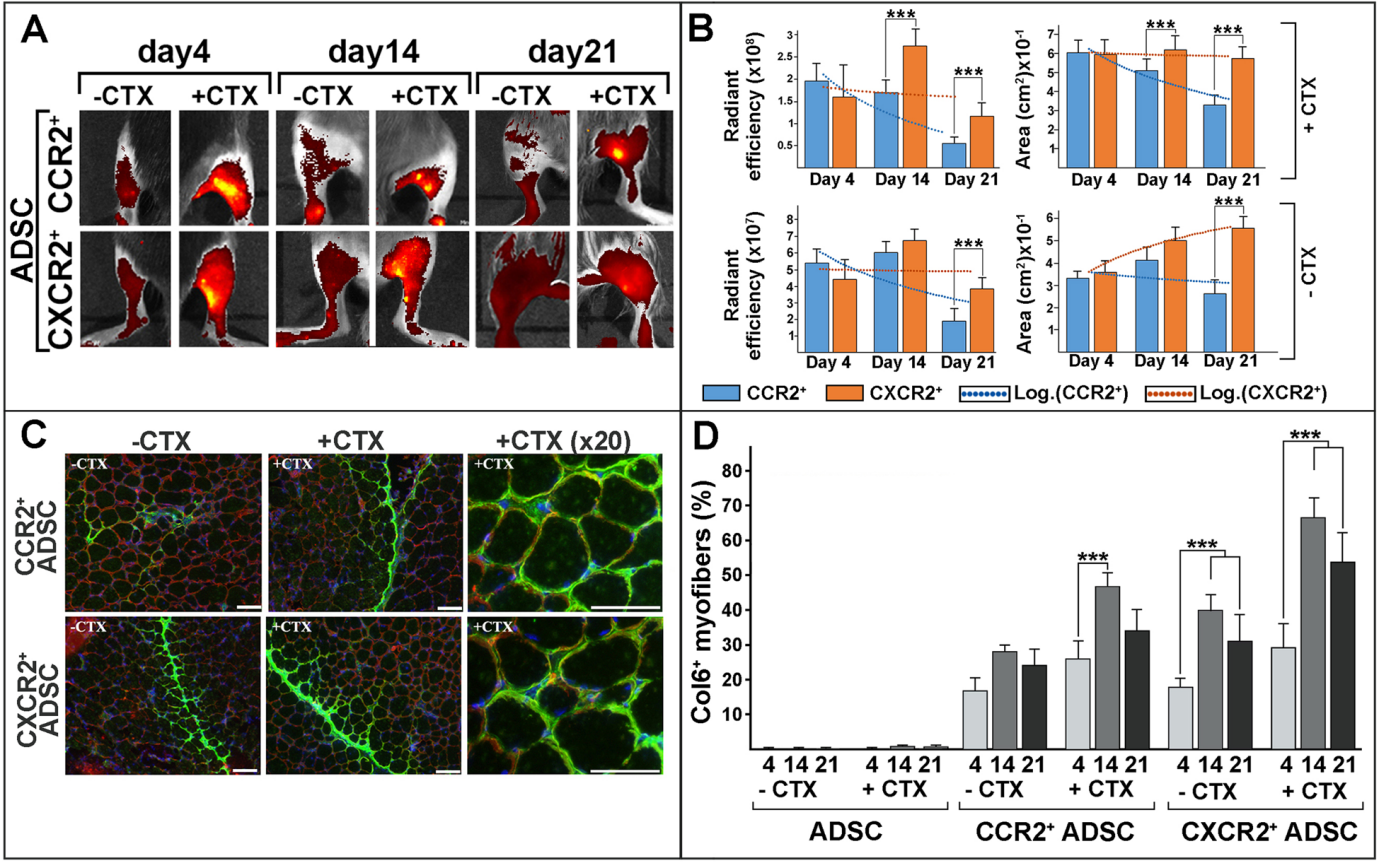
Systemic transplantation of selected ADSC into *Col6a1*^*−/−*^ mice. **a** Representative in vivo images showing the hindlimbs of mice receiving Ccc2-positive ADSC and Cxcr2-positive ADSC transplants under physiological (−CTX) and pro-inflammatory (+CTX) conditions, respectively. IVIS live imaging was performed at 4, 14, and 21 days post-transplant. **b** Quantitative analysis of fluorescence detected by live imaging from differently treated cohorts of mice (*n* = 3 per time point) at 3 time points (as indicated below the columns). Data are presented as radiant efficacy ± SD and as fluorescent area ± SD, respectively. Color-coding for Ccr2-positive ADSC and Cxcr2-positive ADSC is shown in the key. Dotted trend lines illustrate time-dependent changes in differently treated mice, as indicated in the key. Statistically significant differences in Ccr2- and Cxcr2-positive ADSC-treated mice (*p* < 0.05) are indicated with asterisks (***). **c** Immunofluorescent analysis of GCM tissue from untreated and CTX-treated muscle biopsies was performed 21 days after transplantation. Co-localization of the ADSC-donated COL6 and basement membrane-associated type IV collagen was detected with α1(VI) collagen (AlexaFluor^488^, green) and LAMA2 (AlexaFluor^594^, red) antibodies. Images were taken from representative sections at low and high (20x) magnification, respectively. Nuclei were stained with DAPI (blue). Scale bar, 100 μm (low magnification) and 25 μm (high magnification), respectively. **d** Quantitative assessment of COL6-positive myofibers on sections of muscle tissue from mice treated with unselected ADSC, Ccr2-positive ADSC, and Cxcr2-positive ADSC, respectively. Data are presented as the percentage of COL6-positive myofibers per microscopic field ± SD. Time points of tissue collection and treatments are indicted below the columns. Statistical significance (*p* < 0.05) is indicated with asterisks (***)

Comparative proteome analysis of human and mouse CMD samples as well as pairwise matching of identified chemokines and their cognate receptors allowed us to identify few common chemotactic axes, including CXCL7/CXCL1,2,3-CXCR2 and CCL2-CCR2, as potential gradients for targeting of ADSC into CMD muscles. Chemotactic activity of ADSC uniformly expressing CMD muscle-specific chemokine receptors was assessed in a pre-clinical setting using the COL6 deficient mouse model. Reinstatement of COL6 deposition in skeletal muscles was examined after systemic transplantation of selected Ccr2- and Cxcr2-positive ADSC (1.5–2 × 10^6^/injection/mouse) fluorescently labeled with DiOC18 under physiological and CTX-induced inflammatory conditions, respectively. The right GCM was injected with CTX and left GCM was injected with PBS and served as control for natural homing followed by cell transplant 24 h after injury. To compare potential of selected ADSC in normal and muscle-damaged condition, mice were also treated with unselected ADSC in a similar fashion. Live imaging of both CTX-treated and control limbs was performed at 4 (dorsal view), 14, and 21 (ventral view) days after cell transplantation. The experimental animals were concluded 3 weeks after a single transplant. The mice transplanted with heterogeneous ADSC did not show any appreciable signals (data not shown). Contrary, the red fluorescent signals of DiOC18/Ccr2- and DiOC18/Cxcr2-positive ADSC were detectable in all transplanted animals and persisted for the entire study, suggesting the efficient homing and engraftment of cells in the muscle environment under the influence of chemokines in both physiological and CTX-injured conditions (Fig. 5a). Moreover, the detected fluorescent signals were significantly higher in the limbs treated with CTX. Interestingly, the transplantation with Cxcr2-positive ADSC (Cxcl1/Cxcr2, Cxcl2/Cxcr2, and Cxcl5/Cxcr2 axis) resulted in more efficient migration than Ccr2-positive ADSC (Ccl2/Ccr2 axis) (Fig. 5a, b). This is in good agreement with our chemokine protein array profiles showing the significant induction of Cxcl1, Cxcl2, and Cxcl5 chemokines after CTX injury, which are all potent chemoattractants for Cxcr2-positive inflammatory cells. Direct immunofluorescence analysis of muscle biopsies at 21 days after transplant revealed significant presence and wide distribution of DiOC18-ADSC in muscle interstitium (Fig. 5c). Co-immunostaining with antibodies against COL6 and LAMA2, which is an integral component of the muscle basement membrane, showed overlapping fluorescent signals of both proteins at the basement membrane of individual myofibers in both Ccr2- and Cxcr2-positive transplants. Morphometric analysis showed that the number of COL6-labeled myofibers was greater in Cxcr2-positive ADSC transplanted mice (Fig. 5d). Together, these findings strongly suggest that stem cells uniformly expressing specific receptors can be efficiently targeted to the CMD-affected skeletal muscle under influence of the CMD muscle-derived chemotactic signals.

## Discussion

CMD pathogenesis is associated with the lack or dysfunction of the ECM, which provides support and protection from the shearing forces of muscle contraction. We have suggested that ADSC known to produce various ECM molecules, including collagen VI, could be used as cellular therapeutics for this hereditary disorder. Our prior studies on localized transplantation of ADSC into CMD-affected muscle of mice mimicking *Col6a1*-dependent COL6RM confirmed feasibility of ADSC-based therapy. Considering an involvement of the whole body musculature in the pathogenesis of this disorder, systemic approaches are needed for an effective clinical application. The primary challenge in the therapeutic application of stem cells for CMD is the inefficient recruitment of therapeutic cells to the damaged muscles. The precise mechanisms responsible for adult stem cell migration to skeletal muscles in normal and pathological conditions are still largely unknown but recent studies suggested that chemokines are important players in skeletal muscle regeneration [23]. It was shown that experimentally injured skeletal muscles and dystrophic muscles of *mdx* mice are characterized by an inflammatory “molecular signature” in which CC and CXC chemokines are prominent [15, 16]. To date, no sufficient data is available regarding chemotactic signatures in CMD-affected muscles either in patients or in animal models. Chronic inflammation plays an important role in the pathogenesis of CMD. One of the critical features of inflamed muscle tissue is continuous infiltration of muscles with leukocytes under the guidance of chemokines, which define type and degree of inflammation. In this study, we investigated chemotactic gradients of CMD-affected muscle tissues from CMD patients and CMD animal models, and defined chemotactic axes, which could be employed for the targeting of therapeutic ADSC to inheritably weakened muscle tissue.

Comparative proteome analysis indicates that levels of CCL5, CXCL6, CXCL7, CCL2, and GRO are significantly elevated in muscle biopsies from COL6RM and MDC1A patients as compared to control muscle. These data suggest that this set of chemokines is available to provide an appropriate chemotactic gradient to direct therapeutic ADSC to patients’ muscle tissue. All identified molecules are prominent chemotactic factors that activate and attract various inflammatory leukocytes.

In contrast to the CMD patients, proteome analysis of muscle biopsies from *dy*^*w*^ and *Col6a1*^*−/−*^ mice revealed the significant induction of many chemokines. These findings in the *dy*^*w*^ model are consistent with observations that the *dy*^*w*^ model presents with a severe phenotype correlating to the pathology of human MDC1A. This is in contrast to the *Col6a1*^*−/−*^ mouse model, which produces no detectable COL6 protein, but displays a mild phenotypic severity when compared to human UCMD and more similar to the milder BM. Therefore, to explore chemotactic recruitment of ADSC from the systemic compartment, induction of the inflammatory process in the *Col6a1*^*−/−*^ mouse was accomplished through administration of a myonecrotic agent, the *Naja mossambica mossambica venom* CTX, which causes selective damage by targeting myofibrils and inducing inflammation, as previously shown in the same model [22]. After CTX administration, many chemokines identified in LAMA2-deficient muscles were also present in CTX-damaged muscle of *Col6a1*^*−/−*^ mice, suggesting that both diseases share similar chemotactic signals in mice. Rapid induction and sustained expression of identified chemotactic molecules in CTX-injured muscles suggest that chemotaxis could serve as a primary mechanism for continuous and selective recruitment of chemokine receptor-positive inflammatory and therapeutic cell.

Although a precise role of the identified chemotactic molecules in CMD pathology remains to be identified, it is likely that the pattern and the level of specific chemokines in different CMD pre-define inflammatory infiltrate and degree of muscle injury. Our data showing elevated levels of several CC-motif-containing chemokines extend prior studies showing increased expression CCL2, CCL3, and CCL4 in muscle biopsies obtained from animal models and human patients suffering from muscular dystrophy or inflammatory myopathies [23]. Besides playing a role in CMD pathology, some of these chemokines could be attractive for the targeting of therapeutic ADSC from circulation into chronically injured CMD-affected muscles. For example, CCL2-mediated chemotaxis is critical for tissue recruitment of monocytes/ macrophages upon inflammation and infection. It exerts its activity via interaction with CCR2 receptor and plays a pathogenic role in chronic inflammatory diseases, including multiple sclerosis, atherosclerosis, and rheumatoid arthritis [24]. Conversely, CCL2-mediated inflammatory response is essential for repair of acute skeletal muscle injury. *Ccr2*^*−/−*^ mice and mice receiving antibodies against CCL2 show markedly reduced macrophage infiltration in response to acute muscle injuries induced by ischemia or myotoxic agents, and the diminished inflammatory response is accompanied by poor muscle regeneration [25]. CCL5, also known as RANTES (Regulated upon activation, normal T cell expressed, and secreted), plays an essential role in leukocytes (monocytes, macrophages, T cell subsets, DCs, eosinophils, and basophils) homing to the sites of inflammation [26]. It can also stimulate eosinophils to secrete eosinophil cationic protein and stimulate basophils to release histamine. In collaboration with T cell-derived cytokines (e.g., IL-2 and IFNγ), it can also activate NK cell. This chemokine plays an important role in a large number of inflammatory, allergic, autoimmune, or infectious diseases. CCL5 mediates its chemotactic activity through binding to CCR1, CCR3, and CCR5 receptors on the cell surface. As CCR5 serves as HIV-1 co-receptor, a number of inhibitors have been design to block CCR5. However, only a few studies aimed at development of CCL5 inhibitory strategies for different inflammatory disorders were conducted [27]. Our data showing significantly elevated CCL5 level in CMD-affected muscles, suggest that CCL5 could be a novel therapeutic target for CMD and that its inhibition could attenuate CMD-associated inflammation and fibrosis. This view is indirectly supported by our data showing that CCL5 levels are associated with CMD severity with the lowest levels in BM samples and the highest LAMA2 biopsies. Although these observations also support a prognostic value of this chemokine for CMD severity assessment, further evaluation and biostatistical analysis of this chemokines in biopsies and blood samples of a larger cohort of patients will be required. Other upregulated CC-motif containing chemokines including RARRES2 (also known as chemerin), CCL8, CCL9/CCL10, and CCL6 (identified only in rodents) are potent chemoattractants for different leukocytes including monocytes, macrophages, eosinophils, basophils, NK, and T cells. Majority of these ligands are chemotactic to cells expressing CCR1, CCR2, and CCR5 receptors.

Assessment of chemotactic signals in CMD-affected muscles also showed that the majority of significantly upregulated CXC-motif-containing chemokines transmit their chemotactic activity via CXCR1 and CXCR2 chemokine receptors. This group includes GRO family chemokine (CXCL1, CXCL2, CXCL3), CXCL7, and CXCL8. All of them involved in neutrophils, macrophages and others cell of myeloid origin. The functions suggest that these chemokines could not only exacerbate CMD muscle inflammation but also promote its chronicity by constitutively attracting and activating these cells. As shown in models of muscular dystrophies and other myopathies, both neutrophils and macrophages have a capacity to kill muscle cells [28], and their presence was associated with an aggravated of muscle pathology [29]. These data along with our findings are in a good agreement with a recently published study showing that muscles of UCMD patients are significantly infiltrated with M2 macrophages [30]. CXCL12 is another CXC-motif chemokine upregulated in mouse CMD muscles. This molecule is chemotactic for T cells and monocytes during acute inflammatory responses [31]. CXCL12 stimulates migration of monocytes and T-lymphocytes through its receptors, CXCR4, and plays a significant role in regulating migration of both proliferating and terminally differentiated muscle stem cells. Inhibition of this axis was associated with impaired migration and increased apoptosis of skeletal muscle progenitor cells during embryogenesis [32].

Considering proteome data, we suggested that CCR2 and CXCR2 could be the key receptors to direct CMD muscle-specific migration and homing of ADSC. The capacity of systemically administered ADSC to participate in regeneration of skeletal muscle was evaluated using the CTX-induced myonecrosis model with actively ongoing regeneration and remodeling of muscle tissue. Our data clearly demonstrated that both CCR2 and CXCR2 receptors provided directional migration of the systemically administered ADSC into injured muscles. Both, CCR2- and CXCR2-positive ADSC were retained in the muscle for a considerable time (3 weeks of observations), suggesting that transplants were homed to the tissue. However, based on quantitation of live imagings, CXCR2-positive ADSC were twice more effective in homing into muscles than CCR2-positive cells. As illustrated by the trend lines (Fig. 5b), CXCR2 receptor provided sustainable presence of ADSC in muscle tissue. In part, this could be explained by a higher number of upregulated CXCR2 ligands in CTX-injured muscles. This sustainability could also result from different molecular events involved in recruitment of cells from circulation into the tissue. This process involves chemokine-mediated binding of cells to the endothelial lining of the lumen of the blood vessels following extravasation of cells through the vessel wall and migration within the tissue [33]. Considering that CXCR2 ligands are released by endothelial cells of vasodilated blood vessels to recruit leukocytes to the sites of inflammation, it is plausible that extravasation of CXCR2-positive cells is more efficient than CCR2-positive ADSC due to stronger extravasation signals provided by CXCR2 ligands. Moreover, COL6-producing CXCR2-posotive ADSC migrated intramuscularly more efficiently. As illustrated by the trend lines (Fig. 5b), there is a continuous increase in the area covered by CXCR2-positive ADSC. This observation suggests that CXCR2 ligands, which are widely expressed throughout the damaged muscle, also provide sufficient chemotactic signals for intramuscular migration of the transplanted cells. In addition, CXCR4-CXCL12 chemotactic axis may also play an important role in the intramuscular migration of ADSC. Our analysis showed elevated expression of CXC12 in CTX-injured and CDM muscles. Taking together these data with prior observations showing CXCL12-mediated induction of its receptors on ADSC [34], it is plausible that once ADSC are recruited to the damaged muscles, CXCL12-CXCR4 axis could enhance intramuscular migration of the therapeutic cells. This notion is indirectly supported by prior data showing that CXCL12-CXCR4 axis regulates migration of both proliferating and terminally differentiated muscle cells [35].

Significant impact of chemokine receptor-mediated targeting of systemically infused ADSC on the therapeutic effect is also supported by the assessment of the COL6-positive myofibers in differently treated mice. Thus, very few COL6-positive myofibers were found in CTX-injured muscles treated with unselected ADSC, whereas more than 60% of COL6-positive myofibers were found in CTX-injured muscles 14 days after transplantation of CXCR2-positive ADSC (Fig. 5d). Sustainability of COL6 donation to the myomatrix of injured muscles for at least for 21 days of observation period strongly supports the utility of stem cell therapy for COL6RM, which exhibits substantially more severe myopathology than the mouse model.

## Conclusion

Analysis of human and mouse CMD-affected muscles revealed a number of upregulated chemokines that could play an important role in CMD pathology and that CCL5-CCR1/3/5, CCL2-CCR2, and CXCL1/2/7/8-CXCR1,2 chemotaxis axes could be used for the targeting of therapeutic cells to the injured CMD muscles. Comparison between CCR2- and CXCR2-positive ADSC populations demonstrated that both receptors provided muscle-specific homing of therapeutic cells. Moreover, CXCR2-mediated recruitment was more potent in directing ADSC to the injured muscle, in the intramuscular migration, and donation of the therapeutic COL6 protein into the myomatrix. Collectively, our data demonstrated that a systemic administration of stem cells expressing pre-defined set of chemokine receptors could be beneficial to counteract the disease phenotype in CMD-affected muscles. Further mechanistic studies to identify critical factors involved in chemotactic-based directional migration of the stem cells will hopefully pave the way for designing rational approaches toward increasing the disease-site targeting efficiency.

## Supplementary information

**Additional file 1 Table S1**. Patients with confirmed diagnosis of Bethlem myopathy (BM, n=5), Ulrich congenital muscular dystrophy (UCMD, n=8) and Merosin-deficient congenital muscular dystrophy type 1A (MCD1A, n=5) [36–41].

**Additional file 2 Figure S1**. Heat map generated from proteome analysis of human and mouse chemokines reflecting protein expression values in human (A) and mouse (B) muscle biopsies. BM, Bethlem myopathy; UCMD, Ulrich congenital muscular dystrophy; MDC1A, Merosin-deficient congenital muscular dystrophy type 1A.

## Data Availability

All data generated or analyzed during this study are included in this published article. Data sharing is not applicable to this article, as no datasets were generated or analyzed during the current study. However, the data that support the findings of this study are available from the corresponding author upon reasonable request.

## Abbreviations

CMD: Congenital muscular dystrophy
UCMD: Ullrich congenital muscular dystrophy
BM: Bethlem myopathy
COL6: Collagen VI
COL6RM: Collagen VI-related myopathies
LAMA2: Laminin α2
MDC1A: Merosin-deficient congenital muscular dystrophy type 1A
ECM: Extracellular matrix
ADSC: Adipose-derived stem cells
MSC: Mesenchymal stem cells
PBS: Phosphate-buffered saline
BSA: Bovine serum albumin
DMEM/F12: Dulbecco’s modified Eagle medium
FBS: Fetal bovine serum
EDTA: Ethylenediaminetetraacetic acid
FACS: Fluorescence-activated cell sorting
ALP: Alkaline phosphatase
RT-PCR: Reverse-transcriptase polymerase chain reaction
H&E: Hematoxylin and eosin
GCM: Gastrocnemius muscle
CTX: Cardiotoxin
