# Supplementary material for: Congenital muscular dystrophy-associated inflammatory chemokines provide axes for effective recruitment of therapeutic adult stem cell into muscles
Source: Stem Cell Res Ther. 2020 Nov 2;11:463. doi: 10.1186/s13287-020-01979-y (PMC7607684; doi:10.1186/s13287-020-01979-y)
Supplement: Supplementary file 1 — Additional file 1 Table S1. Patients with confirmed diagnosis of Bethlem myopathy (BM, n=5), Ulrich congenital muscular dystrophy (UCMD, n=8) and Merosin-deficient congenital muscular dystrophy type 1A (MCD1A, n=5) [36–41]. [file 13287_2020_1979_MOESM1_ESM.docx]

Table S1. Patients with confirmed diagnosis of Bethlem myopathy (BM, n=5), Ulrich congenital muscular dystrophy (UCMD, n=8) and Merosin-deficient congenital muscular dystrophy type 1A (MCD1A, n=5)

| **Patient** | **Age/Gender** | **Clinical features** | **Mutation** |
| --- | --- | --- | --- |
| #1010 | 40-49/1 | BM; unable to run age 6 yr, DL, FC, severe RI, A | *COL6A2* exon 6 c.802 G>A het; (p.Gly268Ser) [37] |
| #1239 | 30-36/2 | BM; unable to run age 18 yr, FC, mild RI, A | *COL6A2* intron 25 c.1970-3 C>A het; (p.Thr656_Ala698del) [37] |
| #1238 | 10-19/1 | UCMD; floppy at birth, CHD, DL, FC, SC, MV age 11 yr, NA (walked 20 ms-6 yr) | *COL6A1* exon8-intron 8 c.798_804+8del 15 het.; (p.Pro254_Glu268 del) [37] |
| #1240, #799 | 20-29, 10-19/2 | BM; clubfoot, never able to run, diffuse contractures, FC, moderate RI, A | *COL6A2* exon 26 c.2098 G>A; (p.Gly700Ser) [38] |
| #1025 | 1-9/1 | UCMD; floppy at birth, never able to walk, CHD, DL, FC, SC | *COL6A1* exon 9 c.850G>C het; (p.Gly284Arg) |
| #1008 | 1-9/2 | UCMD; floppy at birth, talus of the feet, never able to stand/walk, DL, FC, SC | *COL6A1* exon 9 c.819­_833del het; (p.Pro274_Gly278del) [39] |
| #1001 | 20-29/1 | BM; age 3 difficulty in running & climbing stairs, FC, SC, A | *COL6A2* exon 28 c.2947 2952del6 (p Asp983-2952del6 het); (p. A698A, G699G het) [40] |
| #1055 | 10-19/2 | CMD; floppy at birth, never able to stand/walk, scoliosis, merosin absent |  |
| #1077 | 30-39/1 | CMD, mild; mild muscle weakness, white matter changes, A | *LAMA2* exon 6 c.830C>T het; p.(Ser277Leu) and exon 13 c.1823_1824del; p.(Tyr608*) |
| MCW043.0X | 10-19/1 | CHD, DL, not ambulatory, contractures of neck, shoulder, elbows, hip, knees, FC, SC, respiratory support (BiPap, cough assist | *Col6? no GT* |
| MCW055.0X | 1-9/2 | Contractures, O_2_ at night | *LAMA2*, c.939_940delAT (p.(Cys314Trpfs*3) |
| MCW081.0T | 1-9/2 | BM, CHD, DL, ambulatory w/wheelchair assist, contractures of shoulder, elbows, hips, ankles, toes, FC, SC | *COL6A1, c.1056+1G>A* |
| MCW082.0T | 10-19/2 | CHD, DL, ambulatory, ankle contractures, SC | *COL6A1, c.868G>A, p.(Gly290Arg)(LMNA, lamin A/C)* [41] |
| MCW086.0T | 10-19/2 | DL, ambulatory with walker assist, contractures of neck, elbows knees and ankles | *LAMA2 c.2566delT* |
| MCW087.0T | 10-19/2 | Not ambulatory, SC | *LAMA2 no GT* |
| MCW089.0T | 60-69/1 | No clinical observations | *COL61, c.2642C>T VOUS, p.Ser941Leu \| p.S941L* |
| MCW091.0T | 10-19/1 | DL, not ambulatory, elbow contractures, BiPap respiratory support, SC | *COL6A1, c.887G>T* |

* Description of the clinical features and mutations of patients. BM, Bethlem myopathy; UCMD, Ullrich congenital muscular dystrophy; CMD, congenital muscular dystrophy. Moderate respiratory insufficiency corresponds to a FVC between 50% and 70%; severe respiratory insufficiency corresponds to a FVC less than 50% [42]. A, ambulant; CHD, congenital hip dislocation; DL, distal laxity; FC, finger contractures; MV, nocturnal mechanical ventilation; NA, not ambulant; RI, respiratory insufficiency; SC, skin changes (keloid formation, follicular hyperkeratosis).
