# Supplementary material for: Congenital muscular dystrophy-associated inflammatory chemokines provide axes for effective recruitment of therapeutic adult stem cell into muscles
Source: Stem Cell Res Ther. 2020 Nov 2;11:463. doi: 10.1186/s13287-020-01979-y (PMC7607684; doi:10.1186/s13287-020-01979-y)
Supplement: Supplementary file 2 — Additional file 2 Figure S1. Heat map generated from proteome analysis of human and mouse chemokines reflecting protein expression values in human (A) and mouse (B) muscle biopsies. BM, Bethlem myopathy; UCMD, Ulrich congenital muscular dystrophy; MDC1A, Merosin-deficient congenital muscular dystrophy type 1A. [file 13287_2020_1979_MOESM2_ESM.docx]

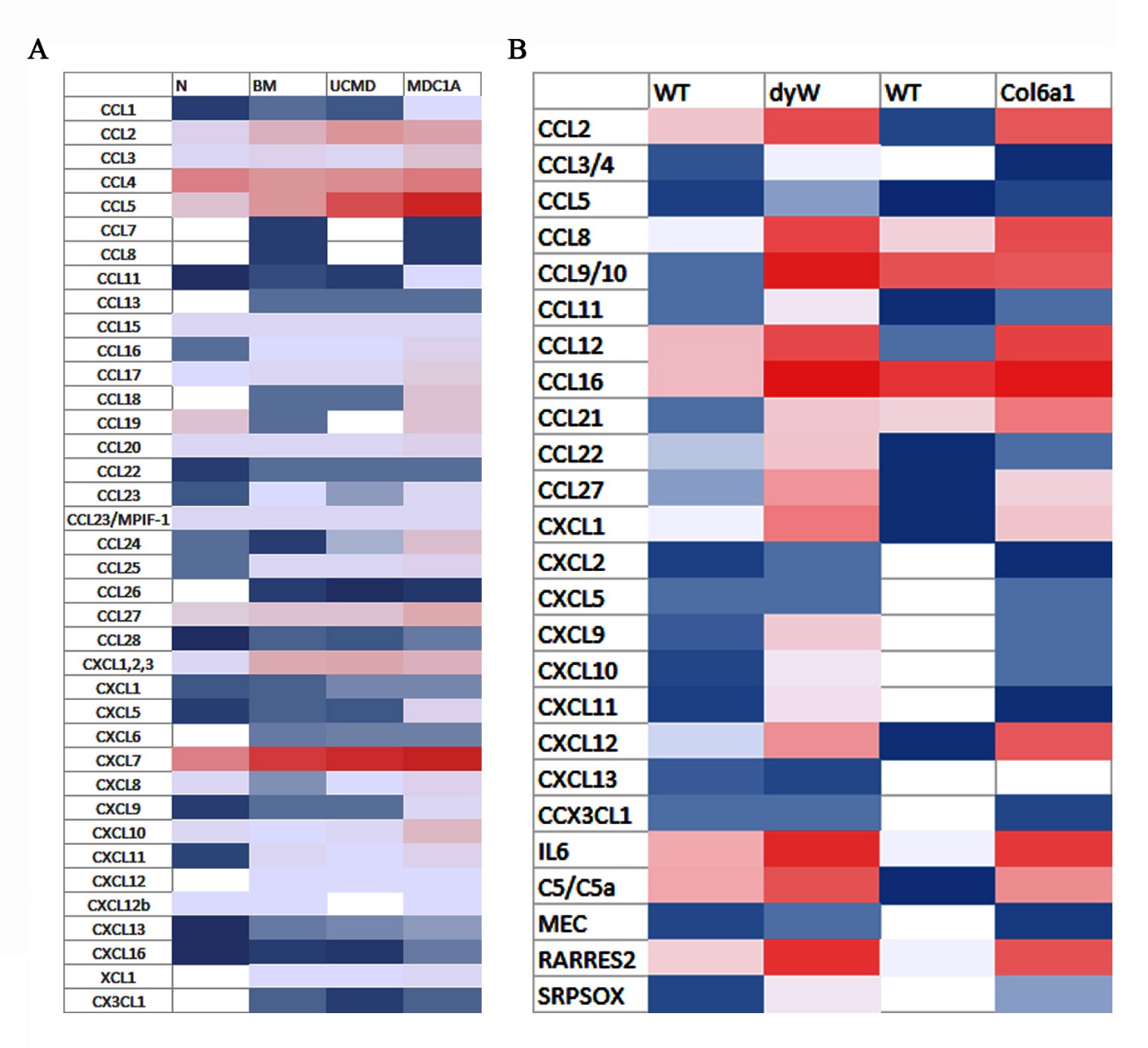


Figure S1. Heat map generated from proteome analysis of human and mouse chemokines reflecting [protein expression](https://en.wikipedia.org/wiki/Gene_expression) values in human (A) and mouse (B) muscle biopsies. BM, Bethlem myopathy; UCMD, Ulrich congenital muscular dystrophy; MDC1A, Merosin-deficient congenital muscular dystrophy type 1A.
